# Connecticut’s Veterans-Directed Care Program Outcomes Compared to HCBS Waiver Participants: HCBS CAHPS Results

**DOI:** 10.1093/geroni/igab046.1396

**Published:** 2021-12-17

**Authors:** Kathy Kellett, Martha Porter, Dorothy Wakefield, Julie Robison

**Affiliations:** 1 UConn Health, Farmington, Connecticut, United States; 2 University of Connecticut, University of Connecticut, Connecticut, United States

## Abstract

Connecticut (CT) Veterans Directed Home and Community Based Services Program (VDC) is an innovative Veterans Administration (VA) services option providing veterans at risk of institutionalization with person-centered consumer-directed long-term services and supports at home. Funded by an Administration for Community Living grant, the CT Department of Aging and Disability Services partnered with the VA, the five CT Area Agencies on Aging, and UConn Health Center on Aging (UConn). UConn researchers conducted the Consumer Assessment of Healthcare Providers and Systems in Home and Community Based Services (HCBS CAHPS) survey with VDC participants (n=36) from October 2019 through March 2020. The standardized, validated HCBS CAHPS survey, which Connecticut administers to individuals in most CT Medicaid HCBS programs, is a universal, cross-disability tool to assess/improve the quality of HCBS programs. Analyses compared VDC participants’ program experiences to survey results from individuals in the Connecticut Home Care Program (CHCP) (for older adults) (n=629), Personal Care Assistance (PCA) (n=282), and Acquired Brain Injury (ABI) (n=327) waiver programs. Notably, more VDC participants (91%) knew who their support broker was, compared to CHCP, ABI, and PCA (82%, 79%, and 72%, respectively) who knew their case manager; 91% of VDC participants gave their support broker the highest rating, compared to 66% to 74% of participants in other programs who rated their case manager. This study provides strong evidence that the CT VDC program is positively impacting veterans and that the AAAs and support brokers are effectively helping them receive the HCBS they need in a consumer-directed way.

